# MicroRNA-30a functions as tumor suppressor and inhibits the proliferation and invasion of prostate cancer cells by down-regulation of SIX1

**DOI:** 10.1007/s13577-017-0170-1

**Published:** 2017-06-01

**Authors:** Qinghuan Zhu, Hongzhi Li, Yingjie Li, Lining Jiang

**Affiliations:** Department of Urinary Surgery, Cangzhou Central Hospital, No. 16 Xinhua Road, Hebei, 061000 People’s Republic of China

**Keywords:** Prostate cancer, miR-30a, SIX1, Proliferation, Invasion

## Abstract

Increasing reports have demonstrated that aberrant expression of microRNAs (miRNAs) is found in multiple human cancers. Many studies have shown that down-regulated level of miR-30a is in a variety of cancers including prostate cancer (PCa). However, the precise mechanisms of miR-30a in PCa have not been well explored. In this study, we investigated the biological functions and molecular mechanism of miR-30a in PCa cell lines, discussing whether it could be a therapeutic biomarker of PCa in the future. We found that miR-30a is down-regulated in PCa tissues and cell lines. Moreover, the low level of miR-30a was associated with increased expression of SIX1 in PCa tissues and cell lines. Up-regulation of miR-30a significantly inhibited proliferation of PCa cells. In addition, invasion of PCa cells was suppressed by overexpression of miR-30a. However, down-regulation of miR-30a promoted cell growth and invasion of PCa cells. Bioinformatics analysis predicted that the SIX1 was a potential target gene of miR-30a. Next, luciferase reporter assay confirmed that miR-30a could directly target SIX1. Consistent with the effect of miR-30a, down-regulation of SIX1 by siRNA inhibited proliferation and invasion of PCa cells. Overexpression of SIX1 in PCa cells partially reversed the effect of miR-30a mimic. In conclusion, introduction of miR-30a dramatically inhibited proliferation and invasion of PCa cells by down-regulating SIX1 expression, and that down-regulation of SIX1 was essential for inhibition of cell growth and invasion of PCa cells by overexpression of miR-30a.

## Introduction

Prostate cancer (PCa), one of the most prevalent malignancies, is a common male malignancy and causes an increase in mortality worldwide every year [1]. Since the growth and metastasis of PCa cells is rapid, PCa is still unmanageable [2]. Although cancer therapies are well developed over the past decades, there is still short of effective therapeutic method for PCa [3]. The precise molecular mechanisms of the pathogenesis of PCa are unclear, which seriously dampens the development of effective treatment for PCa. Therefore, there is an urgent need to gain a better understanding of PCa pathogenesis for improving the present therapeutic management of PCa.

MicroRNAs (miRNAs) are a subset of small (about 22 nucleotides in length), non-coding RNAs [4] which can degrade mRNA or suppress their translation by binding to complementary sequences in the 3′UTRs of targeted mRNAs [5, 6]. MiRNAs regulate a series of cellular processes including cell proliferation, migration, apoptosis, invasion and differentiation [7]. More and more reports demonstrated that miRNAs are dysregulated and involved in progression of many cancers [8]. Recent studies also indicated that multiple miRNAs have been identified to act as tumor suppressors in PCa such as miR-372 [9], miR-382 [10], miR-486-5p [11] and miR-135a-1 [12]. These findings provide a strong basis for the importance of miRNAs in the pathogenesis of PCa and emphasize the implications of miRNAs in diagnosis, therapy, and prognosis of PCa.

Sine oculis homeobox homolog 1 (SIX1), a development-related transcription factor, accelerates cell proliferation and inhibits cell apoptosis in the embryonic development of organs [13, 14]. It has been reported that the high expression of SIX1 is observed in various malignancies including PCa [15–19]. SIX1 could modulate cell cycle and proliferation of tumor cells by transcriptionally regulating the expression of numerous genes. Furthermore, recent studies demonstrated that the increased expression of SIX1 in non-invasive breast cancer cells could accelerate the metastatic capability of the cells [14, 18]. Taken together, these findings suggest that SIX1 may play an important role in the development and progression of caners. However, the roles of SIX1 in PCa are still unknown.

Currently, more and more studies suggest that miR-30a is frequently decreased and functions as a tumor suppressor in several cancers such as colorectal cancer [20], chondrosarcoma [21], hepatocellular carcinoma [22], breast cancer [23] and PCa [24]. It has also been reported that miR-30a can directly target many genes to play critical roles in the development and progression of cancers. However, until now, the direct target of miR-30a in PCa remains unclear. In this study, we also demonstrated that the level of miR-30a was frequently down-regulated in PCa tissues and cell lines, which was consistent with previous study [24]. Overexpression of miR-30a suppressed cell proliferation and invasion of PCa cells. Next, using the on line database, we found that SIX1 might be a direct target of miR-30a. After that, we confirmed that miR-30a could directly target SIX1, a novel tumor suppressor gene, in PCa cells. Moreover, up-regulation of SIX1 reversed the inhibitory effects of miR-30a mimic on PCa cells. Altogether, our data showed important roles for miR-30a in the pathogenesis of PCa and suggested its potential application in its treatment.

## Materials and methods

### Cell culture and human tissues

PNT2 human normal prostate epithelium cell line was purchased from Sigma Aldrich, and cultured with RPMI 1640 media supplemented with 10% fetal bovine serum (FBS, GIBCO, USA), 4 mM glutamine and 1% penicillin and streptomycin (GIBCO, USA). PCa cell lines such as C4-2, 22RV1, DU145, PC3 and RWPE-1 were purchased from American Type Culture Collection (ATCC). These cells were grown in DMEM medium (GIBCO, USA) supplemented with 10% FBS, 1% penicillin and streptomycin at 37 °C in a humidified atmosphere of 5% on 0.1% gelatin-coated culture flasks (Corning, USA). Ten pairs of malignant PCa and their corresponding noncancerous tissues were collected from Cangzhou Central Hospital. The specimens were immediately frozen in liquid nitrogen and then stored at −80 °C for analysis. Prior informed consent was obtained, and the study protocol was approved by the Ethics Committee of Cangzhou Central Hospital.

### MiRNA transfection

To up-regulate or down-regulate the level of miR-30a in PC3 and DU145 cells, cells were transfected with miR-30a mimic and miR-negative control (miR-NC) or miR-30a inhibitor and miR-negative control of inhibitor (anti-miR-NC). One day before transfection, cells were changed to the antibiotic-free medium. After 24 h, cells were transfected with 50 nM miR-30a mimic and miR-NC, 100 nM miR-30a inhibitor and anti-miR-NC using Lipofectamine 3000 reagent (Invitrogen, USA) according to manufacturer’s instructions. The pcDNA3.1 vector and pcDNA-SIX1 were synthesized and purified by Gene-Pharma (Shanghai, China).

### Reverse transcription polymerase chain reaction

Total RNA of PC3 and DU145 was extracted using Trizol reagent (Life Technologies, Carlsbad, CA, USA) for analyzing miRNA and mRNA levels according to the manufacturer’s protocols. For quantification of miR-30a, the TaqMan MicroRNA Reverse Transcription Kit and TaqMan miRNA assay (Applied Biosystems, USA) were used to perform reverse transcription and PCR according to the manufacturer’s instructions. U6 was used as the internal control. The gene expressions of SIX1, PCNA, matrix metalloproteinase (MMP)-2 and MMP-9 were detected using the SYBR Green PCR kits (Qiagen, USA). GAPDH served as an internal control. The following primers were used: PCNA forward, 5′-CCTGCTGGGATATTAGCTCCA-3′, reverse, 5′-CAGCGGTAGGTGTCGAAGC-3′; SIX1 forward, 5′-CTGCCGTCGTTTGGCTTTAC-3′, reverse, 5′-GCTCTCGTTCTTGTGCAGGT-3′; MMP-2, forward: 5′-CTGCGGTTTTCTCGAATCCA-3′ and reverse: 5′-GGGTATCCATCGCCATGCT-3′; MMP-9, forward: 5′-CCCTGGAGACCTGAGAACCA-3′ and revere: 5′-CCACCCGAGTGTAACCATAGC-3′; U6, forward: 5′-CTCGCTTCGGCAGCACA-3′ and reverse: 5′-AACGCTTCACGAATTTGCGT-3′; GAPDH, forward: 5′-GAGTCAACGGATTTGGTCGTATTG-3′ and reverse: 5′-CCTGGAAGATGGTGATGGGATT-3′. U6 snRNA and GAPDH mRNA were used to normalize. Each sample was assessed in triplicate.

### ELISA-BrdU assay

To investigate the effect of miR-30a mimic or inhibitor on cell proliferation of PC3 and DU145 cells, ELISA-BrdU assay was used to detect the cell proliferation using Cell Proliferation ELISA-BrdU Kit (Roche Applied Science, Mannheim, Germany) following the manufacturer’s instruction. Briefly, 5 × 10^3^ cells were seeded in 96-well plate (Corning, USA) and allowed to grow overnight in complete DMEM medium. The medium was then removed and the cells were transfected with miR-30a mimic or inhibitor for 24 h at 37 °C. After 24 h incubation, cells were additionally treated with BrdU labeling solution for the remaining 16 h. After that, culture medium was removed, cells were fixed and DNA was denatured. Cells were incubated with Anti-BrdU-POD solution for 90 min, and then antibody conjugates were removed by washing three times. After incubation with a TMB substrate for 15 min, absorbance at 405 and 490 nm was measured to determine immune complexes.

### Transwell invasion assay

Transwell matrigel invasion assay using Transwell chambers (8-mm pore size; Minipore) precoated with Matrigel (BD Biosciences, Franklin Lakes, NJ, USA) that contained extracellular matrix proteins was used to determined cell invasion. In brief, 1 × 10^5^ cells in 100 μl serum-free DMEM were seeded in the upper chamber, and 600 μl serum-free DMEM was added to the lower chamber. After 24 h incubation at 37 °C in a 5% CO_2_ atmosphere, cells that remained in the upper chamber were removed by cotton swabs and penetrating cells were fixed in methanol, and then stained with 0.1% crystal violet. Then, the membranes were rinsed with 30% glacial acetic acid. Finally, the washing solution was detected at 540 nm for cells counting measurement.

### Measurement of MMP-2 and MMP-9 levels

Enzyme-linked immunosorbent assay (ELISA) kits (USCN, USCN life science, Wuhan, China) was used to determine the levels of MMP-2 and MMP-9 in the culture supernatants based on the manufacturer’s instructions.

### Western blot analysis

PC3 and DU145 cells were washed twice in cold PBS, and then lysed in RIPA lysis buffer (Beyotime Institute of Biotechnology Jiangsu, China) containing protease inhibitor cocktail (Merk, USA). The protein concentration of cell lysates was quantified by BCA Kit (Beyotime Institute of Biotechnology Jiangsu, China), and 50 μg of each of proteins were separated by SDS-PAGE on 10% gels, and then transferred to a polyvinylidene fluoride (PVDF) membrane (Millipore, USA). The membranes were blocked in 5% non-fat milk diluted with TBST at room temperature for 1 h and incubated overnight at 4 °C with primary antibodies such as anti-SIX1 (1:500; Abcam, USA). The membranes were then incubated with a goat anti-rabbit or anti-mouse IgG conjugated to horseradish peroxidase secondary antibody (1:1000; Cell Signaling Technology Inc, MA, USA) for 2 h. The proteins were visualized using ECL-plus reagents (Amersham Biosciences Corp., USA). The density of the bands was measured using the Image J software (USA), and values were normalized to the densitometric values of α-tubulin (1:1000; Sigma, USA) in each sample.

### Luciferase reporter assay

PC3 and DU145 cells were seeded in 24-well plates and incubated for 24 h before transfection. The pMIR-SIX1-3′UTR wild-type or mutant plasmid was cotransfected with miR-30a mimic or miR-NC, and pRL-TK plasmid (Promega, USA) into PC3 and DU145 cells. After transfection for 24 h, luciferase reporter gene assay was implemented using the dual-luciferase reporter system (Promega, USA) following the manufacturer’s protocols. All experiments were performed at least three times.

### Statistical analysis

All statistical analyses were performed using GraphPad Prism 5.0 (GraphPad Software, Inc., USA). Data from each group were expressed as mean ± standard error of the mean (SEM) and statistically analyzed by Student’s *t* test. Differences were considered statistically significant at a *P* value of <0.05.

## Results

### The level of miR-30a is down-regulated in PCa cell lines and tissues

**Fig. 1:**
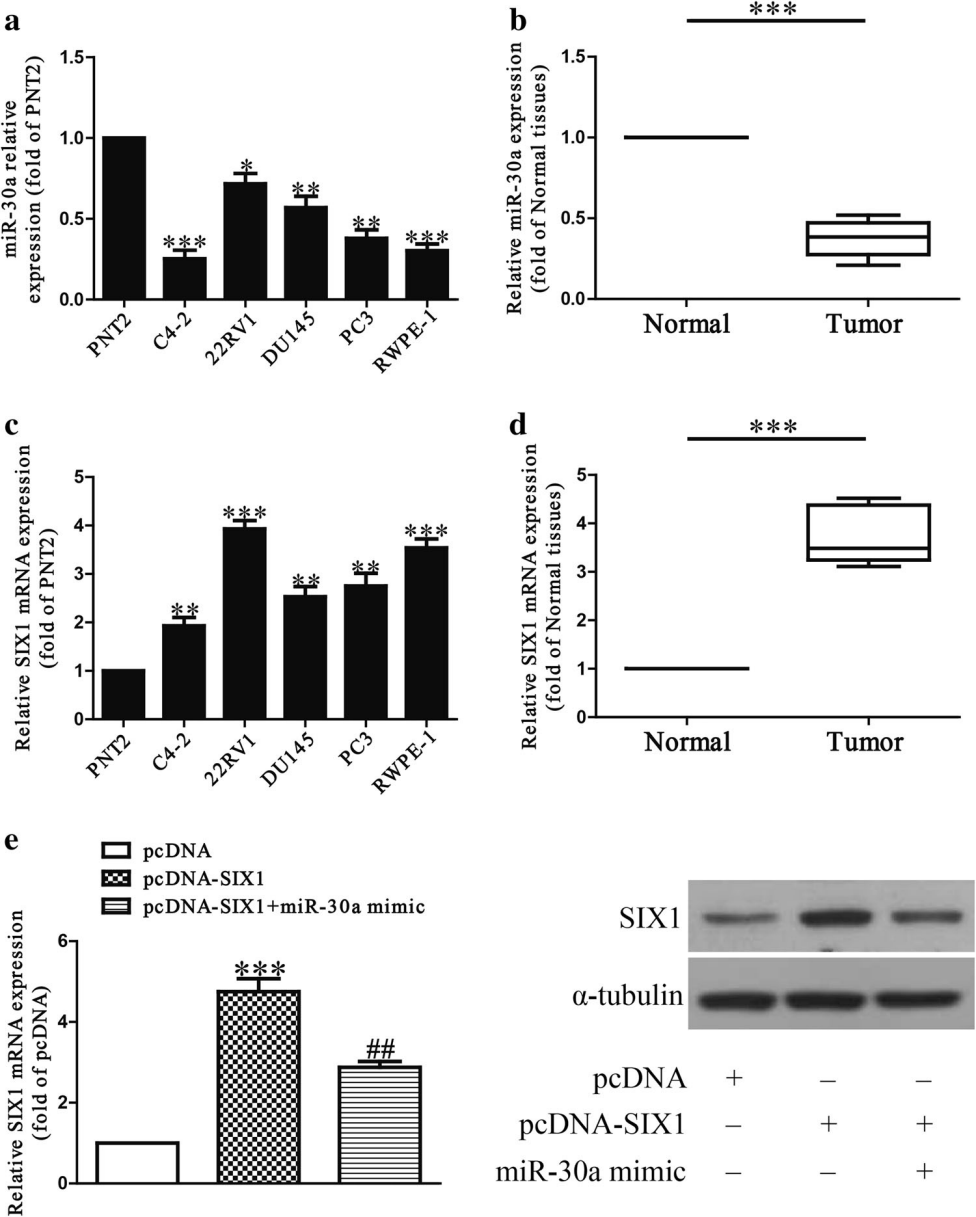
The expression of miR-30a in PCa tissues and cell lines. **a** Relative miR-30a expression levels in PCa tissues and their corresponding adjacent normal tissues. **b** Relative miR-30a level analyzed by qRT-PCR in five PCa cell lines including C4-2, 22RV1, DU145, PC3, RWPE-1 and a human normal prostate epithelium cell line (PNT2) were normalized with U6 snRNA. **c** Relative SIX1 expression levels in PCa tissues and their corresponding adjacent normal tissues. **d** Relative SIX1 mRNA expression analyzed by qRT-PCR in five PCa cell lines including C4-2, 22RV1, DU145, PC3, RWPE-1 and a human normal prostate epithelium cell line (PNT2) were normalized with GAPDH. **e** The SIX1 expression with or without miR-30a mimic analyzed by qRT-PCR and Western blot in in SIX1-overexpressed PC cells. All data are presented as mean ± SEM, *n* = 6. *P < 0.05, **P < 0.01, ***P < 0.001 vs. PNT2 or normal tissues or pcDNA; ^##^ *P* < 0.01 vs. pcDNA-SIX1

It has been reported that miR-30a was down-regulated in multiple cancers, including PCa [20–24]. In this study, the level of miR-30a was detected by qRT-PCR in a human normal prostate epithelium cell line (PNT2) and five PCa cell lines including C4-2, 22RV1, DU145, PC3 and RWPE-1. Our results showed that the level of miR-30a was evidently down-regulated in these five PCa cell lines compared to that in PNT2 (Fig. 1a). Moreover, the level of miR-30a in the PCa tissues was significantly lower in comparison to the adjacent tissues (Fig. 1b). Next, the bioinformatics analysis showed that SIX1 was predicted to be a direct target of miR-30a. So we detected the mRNA level of SIX1 in five PCa cell lines and tissues, respectively. The results indicated that the expression of SIX1 was evidently up-regulated in all PCa cell lines compared to that in PNT2 at mRNA level (Fig. 1c). And SIX1 expression in PCa tissues was also significantly increased compared to adjacent normal tissues (Fig. 1d). For further study, we checked the expression of SIX1 with or without miR-30a mimic in SIX1-overexpressed PC cells (pcDNA-SIX1), to confirm the direct association of SIX1 with miR-30a. Our results showed that miR-30 mimic could significantly decrease the SIX1 expression at mRNA and protein levels in SIX1-overexpressed PC cells (Fig. 1e). From the above data, we predicted that SIX1 might be negatively regulated by miR-30a.

### MiR-30a inhibited cell proliferation of both PC3 and DU145 cells

**Fig. 2:**
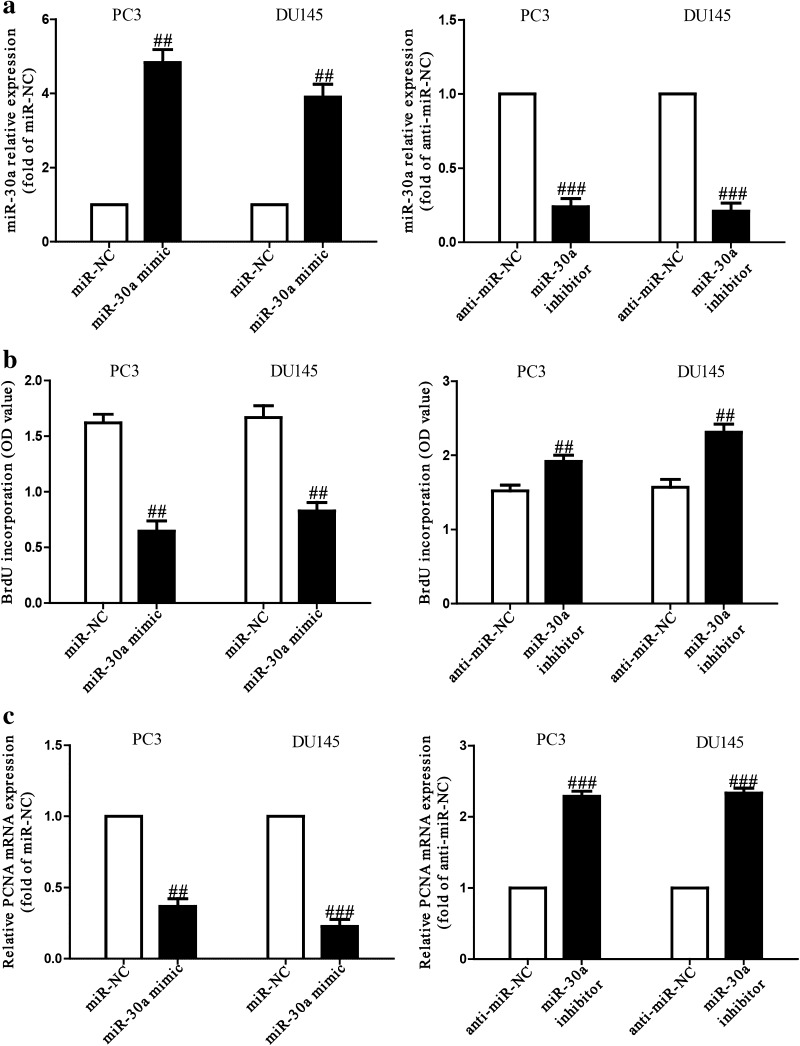
Effects of miR-30a on cell proliferation in PC3 and DU145 cells. PC3 and DU145 cells were transfected with miR-30a mimic or inhibitor for 24 h. **a** The levels of miR-30a in PC3 and DU145 cells were determined by qRT-PCR. **b** Cell proliferation was assessed by BrdU-ELISA assay. **c** The mRNA level of PCNA was determined by Western blot. GAPDH was detected as a loading control. All data are presented as mean ± SEM, *n* = 6. ^##^ *P* < 0.01, ^###^ *P* < 0.001 vs. miR-NC or anti-miR-NC

Since the level of miR-30a was significantly down-regulated in multiple cancers, we believed that miR-30a could act as a suppressor of cell proliferation. After transfection with miR-30a mimic or inhibitor, the qRT-PCR analysis showed that the level of miR-30a was dramatically up-regulated or down-regulated in miR-30a mimic or inhibitor group compared to miR-NC or anti-miR-NC group (Fig. 2a). Our results demonstrated that we efficiently increased or decreased miR-30a expression in PC3 and DU145 cells. To determine the role of miR-30a in proliferation of PCa cells, the results from Brdu-ELISA assay demonstrated that overexpression of miR-30a dramatically inhibited the proliferation of PC3 and DU145 cells, whereas knockdown of miR-30a promoted PCa cell proliferation (Fig. 2b). To further confirm this result, we detected the expression of PCNA protein. We found that miR-30a mimic could evidently reduce the expression of PCNA, and miR-30a inhibitor had the reverse effect on PCNA expression (Fig. 2c).

### Effect of miR-30a on the invasion of PCa cells

**Fig. 3:**
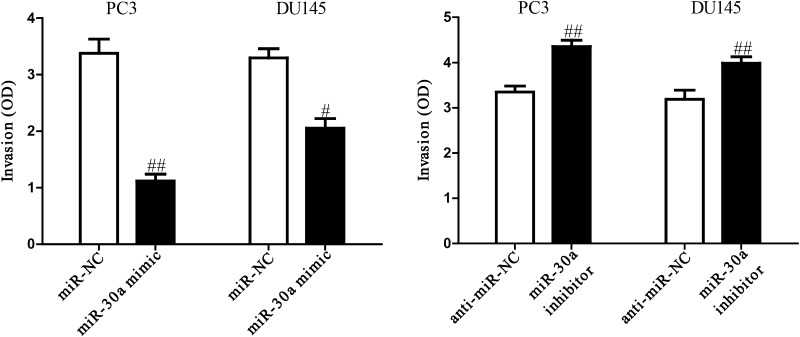
The effects of miR-30a on invasion in PC3 and DU145 cells. **a** The invasion of PC3 and DU145 cells transfected with miR-30a mimic or miR-NC was assessed by Transwell assay. **b** The invasion of PC3 and DU145 cells transfected with miR-30a inhibitor or anti-miR-NC was assessed by Transwell assay. All data are presented as mean ± SEM, *n* = 6. ^#^ *P* < 0.05, ^##^ *P* < 0.01 vs. miR-NC or anti-miR-NC

To investigate the role of miR-30a in invasion of PCa cells, we evaluated the invasive capacities of PC3 and DU145 cells transfected with miR-30a mimic or inhibitor by Transwell assays. Our results showed that the invasive capability of PC3 and DU145 cells was remarkably inhibited in miR-30a mimic group compared to miR-NC group (Fig. 3a), but was evidently enhanced in miR-30a inhibitor group (Fig. 3b). These data confirmed that miR-30a might play a critical role in inhibiting invasion of PCa cells.

### Effects of miR-30a on secretions and expressions of MMP-2 and MMP-9 in PCa cells

**Fig. 4:**
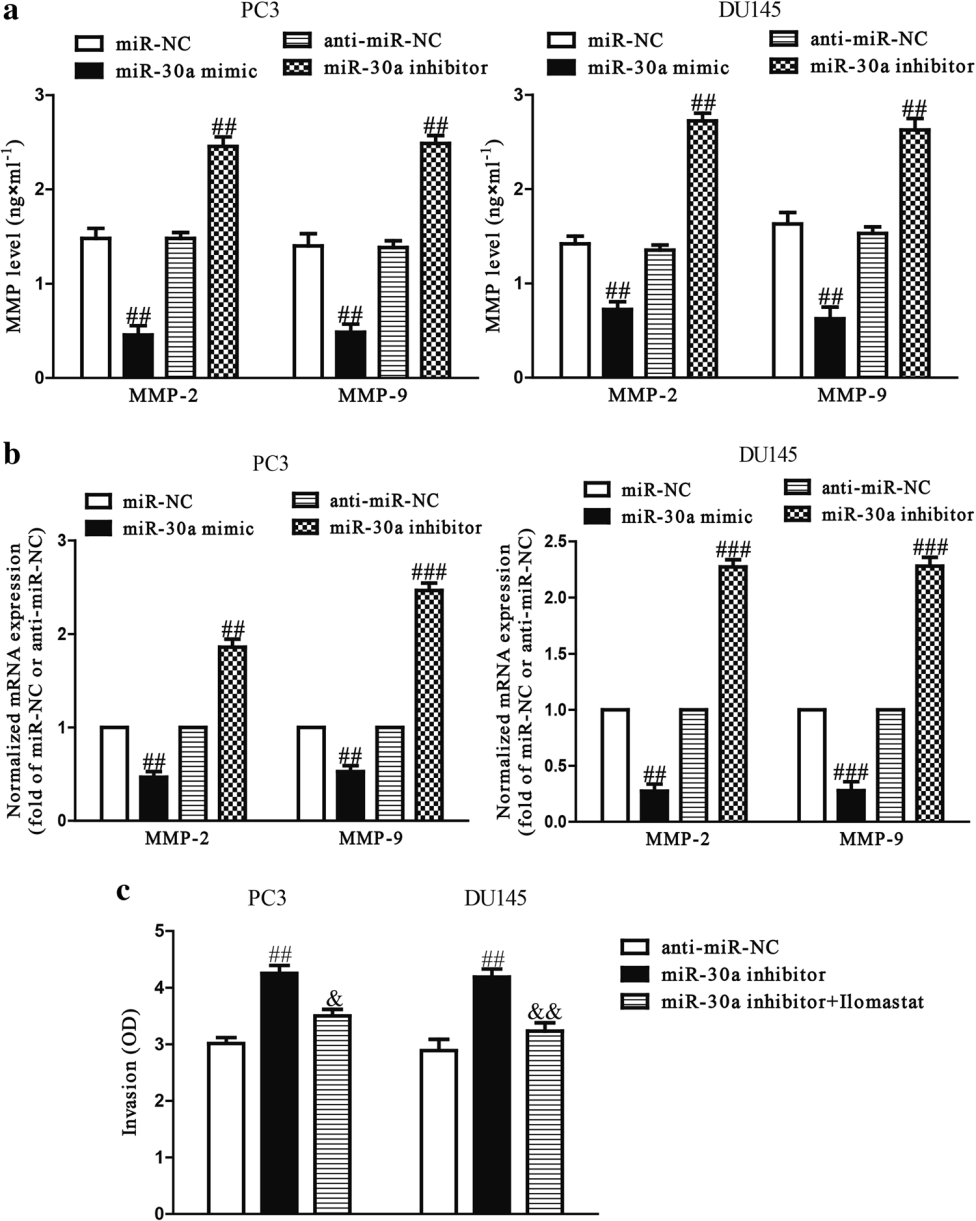
Overexpression of miR-30a suppressed expressions and secretions of MMP-2 and MMP-9. PC3 and DU145 cells were transfected with miR-30a mimic or inhibitor. **a** Levels of MMP-2 and MMP-9 were detected in the culture supernatants of cultured PC3 and DU145 cells by ELISA assay. **b** The mRNA levels of MMP-2 and MMP-9 were examined by qRT-PCR. **c** The invasion of PC3 and DU145 cells transfected with miR-30a inhibitor and treated with Ilomastat (a MMP inhibitor, 50 μM) was assessed by Transwell assay. All data are presented as mean ± SEM, *n* = 6. ^##^ *P* < 0.01, ^###^ *P* < 0.001 vs. miR-NC or anti-miR-NC; ^&^ *P* < 0.05, ^&&^ *P* < 0.01 vs. miR-30a inhibitor

Since MMPs are closely correlated with cell invasion, we detected the expressions of MMP-2 and -9, after transfection with miR-30a mimic or inhibitor. The ELISA assay demonstrated that secretions of MMP-2 and MMP-9 in the culture supernatants were significantly reduced by up-regulation of miR-30a in PC3 and DU145 cells, and evidently enhanced by down-regulation of miR-30a (Fig. 4a). Moreover, we further determined the mRNA levels of MMP-2 and MMP-9 by qRT-PCR. After transfection with miR-30a mimic, the mRNA levels of MMP-2 and MMP-9 were distinctly reduced compared to miR-NC group, and miR-30a inhibitor enhanced the mRNA levels of both compared to anti-miR-NC (Fig. 4b). Our data indicated that down-regulated expressions of MMP-2 and MMP-9 might be a potential mechanism contributed to the inhibitory effect of miR-30a mimic on the invasion of PC3 and DU145 cells. To confirm this result, we used Ilomastat (a MMP inhibitor, 50 μM) to treat the PCa cells transfected with miR-30a inhibitor. The data indicated that Ilomastat dramatically suppressed miR-30a inhibitor-induced the invasion of PC3 and DU145 cells (Fig. 4c).

### SIX1 is a direct target of miR-30a in PCa cells

**Fig. 5:**
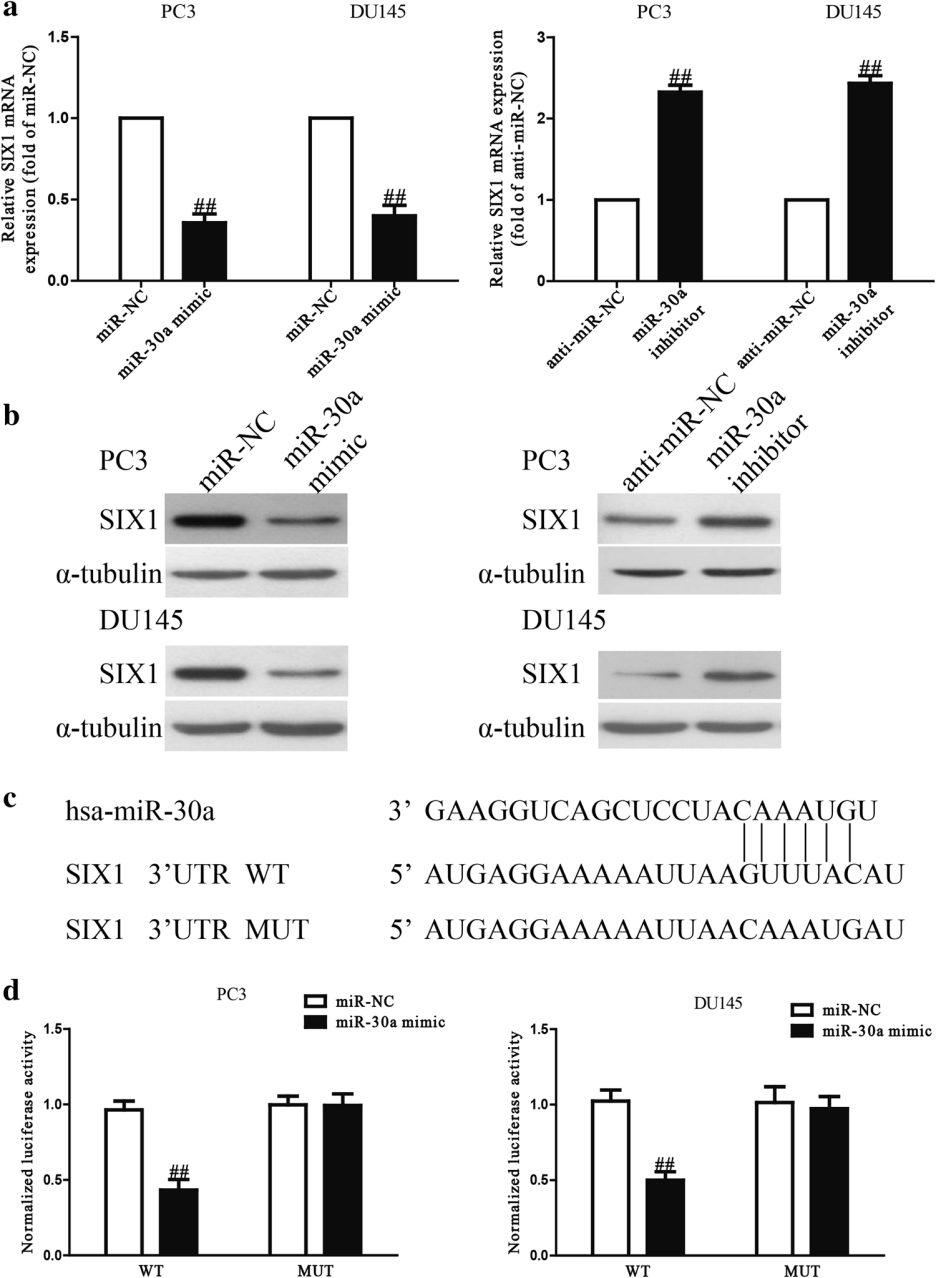
SIX1 was a direct target of miR-30a. **a** The mRNA level of SIX1 was determined by qRT-PCR in PC3 and DU145 cells transfected with miR-30a mimic or inhibitor, respectively. **b** The protein expression of SIX1 was determined by Western blot in PC3 and DU145 cells transfected with miR-30a mimic or inhibitor, respectively. **c** Schematic representation of SIX1 3′UTRs showing putative miRNA target site. **d** The analysis of the relative luciferase activities of SIX1-WT, SIX1-MUT in PCa cells. All data are presented as mean ± SEM, *n* = 6. ^##^ *P* < 0.01, ^###^ *P* < 0.001 vs. miR-NC or anti-miR-NC

Since TargetScan 6.2 predicted that SIX1 was a binding target of miR-30a, we determined the expression of SIX1 by qRT-PCR and Western blotting at mRNA and protein levels in PC3 and DU145 cells transfected with miR-30a mimic or inhibitor. The mRNA and protein levels of SIX1 were prominently decreased after transfection with miR-30a mimic, and were increased after transfection with miR-30a inhibitor (Fig. 5a, b). Next, luciferase reporter assay had confirmed that miR-30a directly targeted SIX1. SIX1 3′-UTR was cloned into a luciferase reporter vector and the putative binding site of miR-30a in the SIX1 3′-UTR was mutated (Fig. 5c). The data exhibited that overexpression of miR-30a obviously inhibited the luciferase activity of pMIR-SIX1 3′-UTR WT (Fig. 5d). Mutation of the binding site of miR-30a in the SIX1 3′-UTR abolished the effect of miR-30a mimic, which suggested that SIX1 was directly and negatively regulated by miR-30a.

### MiR-30a suppressed cell proliferation and invasion of PCa cells through inhibition of SIX1

**Fig. 6:**
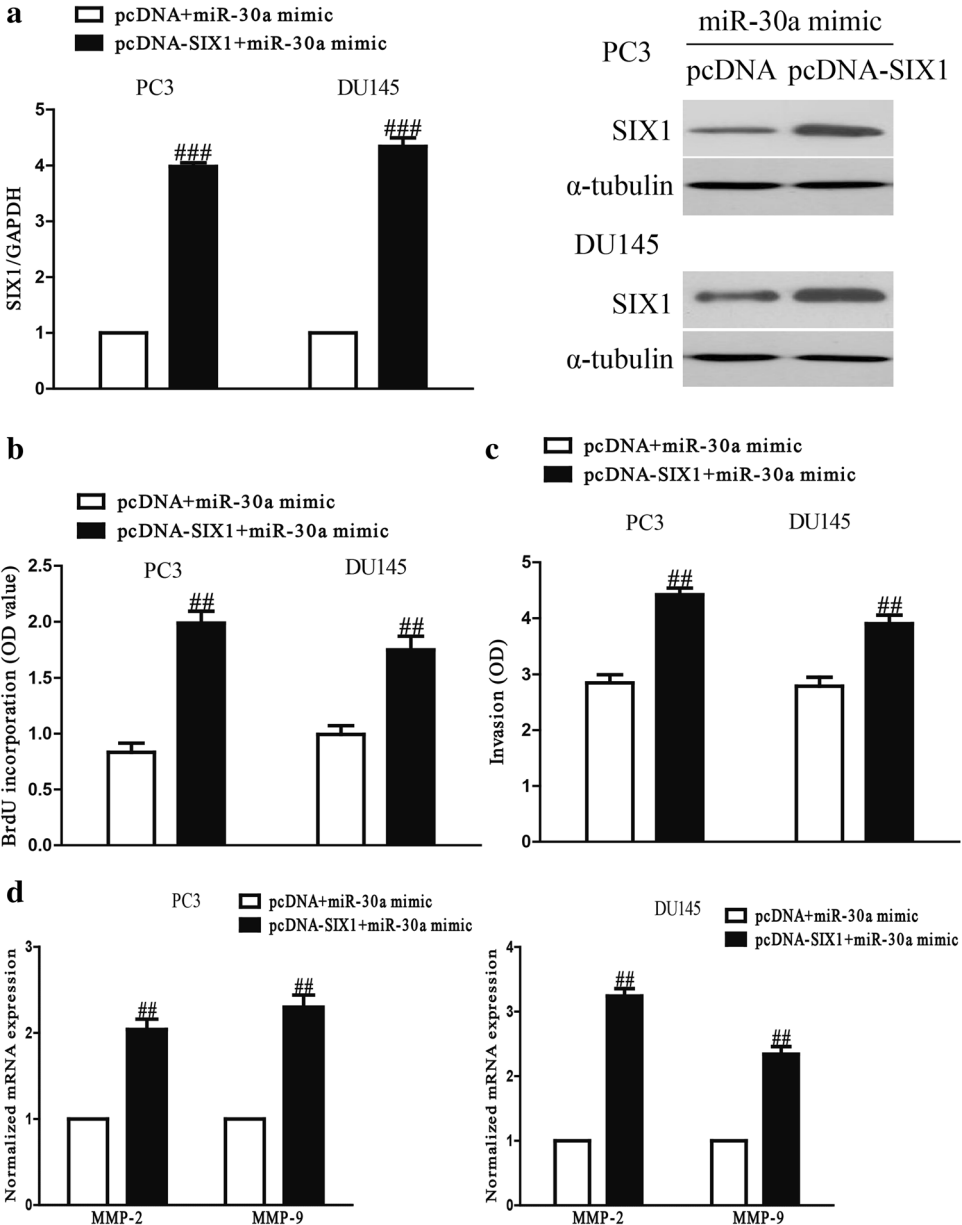
Overexpression of SIX1 partially rescued miR-30a-inhibited cell proliferation and invasion in PCa cells. PC3 and DU145 cells were transfected with either miR-30a mimic with or without pcDNA-SIX1 vector. **a** The mRNA and protein levels of SIX1 were determined by qRT-PCR and Western blot, respectively. **b** Cell proliferation was assessed by BrdU-ELISA assay. **c** The invasion of PC3 and DU145 cells was assessed by Transwell assay. **d** The expressions of MMP-2 and MMP-9 were determined by qRT-PCR in PC3 and DU145 cells, respectively. All data are presented as mean ± SEM, *n* = 6. ^##^ *P* < 0.01, ^###^ *P* < 0.001 vs. miR-30a mimic + pcDNA

To confirm whether miR-30a inhibited the proliferation and invasion of PCa cells through SIX1-dependent mechanism, we cotransfected PC3 and DU145 cells with miR-30a mimic and pcDNA-SIX1 vector. We found that SIX1 expression was significantly increased after transfection with miR-30a and pcDNA-SIX1 compared with miR-30a and pcDNA vector in both PC3 and DU145 cells (Fig. 6a). Data from Brdu-ELISA assay showed that up-regulation of SIX1 in cells transfected with the miR-30a mimic promoted the proliferation of PCa cells (Fig. 6b). The Transwell assay indicated that up-regulation of SIX1 could reverse the inhibitory effect of miR-30a mimic on invasion of PCa cells (Fig. 6c). Next, introduction of SIX1 increased the expressions of MMP-2 and MMP-9 at mRNA level in PC3 and DU145 cells after transfection with miR-30a mimic (Fig. 6d). Therefore, our results clearly demonstrated that miR-30a inhibited cell proliferation and invasion of PCa cells by down-regulation of SIX1, and that knockdown of SIX1 was essential for the miR-30a mimic-induced inhibition of cell proliferation and invasion in PCa cells.

## Discussion

More and more evidence demonstrated that miRNAs can play critical roles in regulating the progression of PCa [25–27]. Therefore, it is of great importance to identify prostate cancer-related miRNAs for use as biomarkers for diagnosis and treatment. As a main tumor suppressor, miR-30a was found to be reduced in many human cancers such as colorectal cancer, chondrosarcoma, hepatocellular carcinoma, breast cancer and PCa [20–24]. The precise mechanism of miR-30a in PCa remained unclear. Therefore, in this study, we were aimed to elucidate the biological functions and its mechanism of miR-30a in PCa. Our data showed that miR-30a was significantly decreased in PCa cell lines and tissues. Based on these results, we speculated that miR-30a might be a potential anti-oncogene in PCa, which was consistent with previous study [24]. As expected, up-regulation of miR-30a inhibited proliferation and invasion of PCa cells. Our findings indicated that miR-30a played important roles in regulating proliferation and invasion of PCa and might be a possible diagnostic and predictive biomarker.

Afterwards, we studied the precise molecular mechanism of miR-30a on inhibition of proliferation and invasion of PCa cells. In this study, we confirmed that miR-30a directly targeted SIX1 using qRT-PCR, Western blotting and luciferase reporter assay. Importantly, we also demonstrated that introduction of SIX1 partly reversed the inhibitory effects of miR-30a mimic on proliferation and invasion of PCa cells. Collectively, we concluded that miR-30a played important roles in inhibition of proliferation and invasion of PCa cells, partially by down-regulating expression of SIX1.

Here, Brdu-ELISA assays showed that overexpression of miR-30a could result in remarkable inhibition of proliferation of PC3 and DU145 cells. qRT-PCR analyses also showed that the expression of PCNA, a proliferative marker, was obviously reduced in cells transfected with miR-30a mimic compared to cells transfected with miR-NC. However, miR-30a inhibitor could evidently accelerate proliferation of PC3 and DU145 cells compared with anti-miR-NC group. In addition, Transwell assay showed that overexpression or knockdown of miR-30a dramatically inhibited or promoted the invasion of PC3 and DU145 cells compared with miR-NC or anti-miR-NC group, respectively. Moreover, to further study the effect of miR-30a on invasion of PCa cell, we determined the expressions of MMP-2 and MMP-9 in PC3 and DU145 cells transfected with miR-30a mimic or inhibitor. One of the key molecular steps in the process of invasion is degradation of extracellular matrix (ECM) components by proteolytic enzymes [28]. MMPs are known to be overexpressed as normal mucosa progresses to adenomas and carcinomas [29]. High activity of MMPs in adenomas is considered as a biomarker of early tumorigenesis [30]. It is well known that MMP-2 and -9 are the major ECM-degrading enzymes, once produced in the tumoral environment, can mainly degrade collagen IV that is a main component of basement membranes compromising the basement membranes integrity [31]. It turned out that overexpression of miR-30a could markedly inhibit invasive ability of PCa cells by dramatically decreasing the expressions of MMP-2 and MMP-9, whereas miR-30a inhibitor had the opposing effect on expressions of EMT markers. Moreover, MMP inhibitor could block the auxo-action of miR-30a inhibitor on invasion of PCa cells.

Many studies have indicated that SIX1, an important oncogene, was found to be overexpressed in various cancers including pancreatic, breast ovarian and cervical cancers [17, 32–34]. In this study, SIX1 was found to be up-regulated in PCa cells and tissues. In vitro, overexpression of miR-30a significantly decreased SIX1 mRNA and protein expression compared to controls, whereas knockdown of miR-30a lead to increased SIX1 expression. Moreover, fluorescent reporter assays confirmed that miR-30a directly bound to the SIX1 3′-UTR region, suggesting SIX1 as a direct target of miR-30a. Besides, restoration of SIX1 reversed the inhibitory effects of miR-30a mimic, suggesting that SIX1 might play an important role in progression and invasion of PCa. Hence, our findings indicated that miR-30a inhibited cell proliferation and invasion, at least partly, through regulation of SIX1.

In conclusion, we have demonstrated that relative miR-30a expression was dramatically reduced in PCa tissues and cells. Up-regulation of miR-30a inhibited proliferation, invasion and EMT of PCa cells by directly down-regulation of SIX1. This novel miR-30a/SIX1 axis might provide new insights into the molecular mechanisms underlying progression and metastasis of PCa, and overexpression of miR-30a might be a possible therapeutic strategy for the therapy of PCa in the future.
